# Preclinical Assessment of Adjunctive tPA and DNase for Peritoneal Dialysis Associated Peritonitis

**DOI:** 10.1371/journal.pone.0119238

**Published:** 2015-03-05

**Authors:** Amanda L. McGuire, Sophia C. Bennett, Sally M. Lansley, Natalia D. Popowicz, Julius F. Varano della Vergiliana, Daniel Wong, Y. C. Gary Lee, Aron Chakera

**Affiliations:** 1 Translational Renal Research Group, Harry Perkins Institute of Medical Research, Perth, Australia; 2 University of Western Australia, School of Medicine and Pharmacology, Perth, Australia; 3 Pleural Disease Unit, Lung Institute of Western Australia, Centre for Asthma, Allergy Respiratory Research, School of Medicine and Pharmacology, Perth, Australia; 4 Department of Anatomical Pathology, PathWest Laboratory Medicine WA, QEII Medical Centre, Perth, Australia; 5 Respiratory Department, Sir Charles Gairdner Hospital, Perth, Australia; 6 Renal Department, Sir Charles Gairdner Hospital, Perth, Australia; University of California Merced, UNITED STATES

## Abstract

A major complication of peritoneal dialysis is the development of peritonitis, which is associated with reduced technique and patient survival. The inflammatory response elicited by infection results in a fibrin and debris-rich environment within the peritoneal cavity, which may reduce the effectiveness of antimicrobial agents and predispose to recurrence or relapse of infection. Strategies to enhance responses to antimicrobial agents therefore have the potential to improve patient outcomes. This study presents pre-clinical data describing the compatibility of tPA and DNase in combination with antimicrobial agents used for the treatment of PD peritonitis. tPA and DNase were stable in standard dialysate solution and in the presence of antimicrobial agents, and were safe when given intraperitoneally in a mouse model with no evidence of local or systemic toxicity. Adjunctive tPA and DNase may have a role in the management of patients presenting with PD peritonitis.

## Introduction

More than 10% of the population has chronic kidney disease (CKD), with 1 in 3 adults at risk of developing CKD during their lives [1]. In Australia, more than 2000 people reach end stage kidney disease and commence renal replacement therapy each year [2]. Of the available dialysis modalities, peritoneal dialysis (PD) is used by over 20% of patients, and may be the only option in remote locations [2]. As PD is usually performed in the patient’s place of residence, potential benefits include reduced cost (when compared to haemodialysis) and improved quality of life [3–6].

A major complication of PD is the development of PD-associated peritonitis, primarily caused by bacterial infections within the abdomen. The definition of PD peritonitis has been standardized and requires two or more of the following criteria: cloudy dialysate fluid and/or abdominal pain and/or fever, dialysate white cell count of >100/μL with >50% neutrophils, or positive culture of dialysate fluid [7]. PD peritonitis occurs approximately once in every 19–28 patient months on treatment [2], and is associated with reduced modality and patient survival [8–11]. In Australia, peritonitis is the cause of technique failure in approximately 20% of patients, requiring them to transfer to haemodialysis [2].

The acute inflammatory response caused by peritonitis is orchestrated by mesothelial cells that line the abdominal cavity, and is characterised by the accumulation of neutrophils and pro-inflammatory cytokines. Release of fibrin is prominent [12], and in conjunction with DNA liberated from bacteria and immune cells, can contribute to the formation of adhesions and biofilms. These may provide physical protection for bacteria from the host immune response and reduce the effectiveness of antibiotic treatment, resulting in refractory, recurrent or relapsing infections [13–18].

Several case reports have documented the resolution of recalcitrant infections in patients with PD peritonitis treated with intraperitoneal fibrinolytic agents [19–21]. The presumed mechanism of action is disruption of protected microenvironments created by fibrin deposition, facilitating the eradication of infecting organisms. Intra-abdominal instillations of fibrinolytics have also been given to reduce adhesions following intra-abdominal surgery and to treat malfunction of uninfected PD catheters, with no evidence of adverse side effects or alterations of systemic coagulation parameters [22, 23].

Empyemas are an accumulation of pus, extracellular DNA and bacterial components in the mesothelial cell-lined pleural cavity surrounding the lungs. The high viscosity of the empyema fluid makes treatment challenging [24–28], and leads to death or surgical intervention in more than 30% of patients [29]. Breaking down extracellular DNA and fibrin, to reduce fluid viscosity and biofilm using intrapleural administration of deoxyribonuclease (DNase) and tPA has recently been demonstrated in a randomized controlled trial to improve outcomes for patients with empyema compared to the use of tPA or DNase alone [29]. This is the largest trial to date to report the instillation of tPA and/or DNase into a mesothelial space and was not associated with any increase in serious or non-serious side effects when compared to placebo [29]. The combination of tPA and DNase has not been previously been investigated as an adjunct for the treatment of peritonitis.

We report a series of pre-clinical investigations to assess the feasibility of administering tPA and DNase in combination with empirical antibiotic therapy in a standard peritoneal dialysis solution for the treatment of peritoneal dialysis-associated peritonitis. We demonstrate that tPA and DNase are compatible with dialysate, that the biological activity of these agents is preserved in the presence of common antimicrobial agents, that the presence of tPA and DNase does not reduce the efficacy of these agents and that administration of these agents in an animal model is not associated with toxicity. Further investigation of tPA and DNase as adjunctive therapy in the management of patients on peritoneal dialysis with peritonitis may be warranted.

## Materials and Methods

### Dialysate Solutions

All experiments were carried out using sterile dialysate solution (CAPD/DPCA 19, 2.3% Glucose, Fresenius Medical Care Australia, Milsons Point, New South Wales, Australia). Sterile dialysate was stored at room temperature and all dialysate solutions were prepared fresh each day.

### tPA, DNase and LPS

tPA (Actilyse Alteplase (rt-PA), rch powder for Injection, 10 mg, Boehringer Ingelheim, New South Wales, Australia) was prepared according to the manufacturer’s instructions by reconstituting the 10 mg vial of tPA with the supplied 10 mL of water for injections. The 1 mg/mL solution of tPA was stored in 300 μL aliquots at -20°C and thawed at room temperature immediately before use. DNase (Pulmozyme Inhalation, Dornase alfa (rch), 2500 U (2.5 mg), Roche, Sydney, Australia) was obtained in ready-to-use ampoules at 1 mg/mL and was stored at 4°C. The final concentrations of tPA (5 μg/mL) and DNase (2.5 μg/mL) correspond to the 10 mg tPA and 5 mg DNase given to patients in 2 L of dialysate solution, equivalent concentrations to those used in the pleural study [29]. Lipopolysaccharide (LPS; Sigma-Aldrich, New South Wales, Australia) was prepared in sterile PBS at a concentration of 2 mg/mL.

### Biochemical Analysis of Dialysate

Samples (5 mL aliquots) of dialysate alone, dialysate containing 5 μg/mL tPA and 2.5 μg/mL DNase and dialysate containing 5 μg/mL tPA and 2.5 μg/mL DNase as well as therapeutic concentrations of various antimicrobial agents were sent to the PathWest Laboratory Medicine WA (Nedlands, Western Australia, Australia) for biochemical analyses and electrolytes were measured, on the Siemens Advia 2400 analyser (Siemens Medical Solutions Pty. Ltd, Bayswater, Victoria, Australia). Samples were analysed at baseline, and after six hours at 37°C. In addition to biochemical analyses, samples were assessed for the formation of precipitants by visual inspection and spectrophometric analysis (of 100 μL aliquots) at 650 nm using a SpectraMax Plate Reader (Molecular Devices, CA, USA).

### Bacterial Isolates

Bacterial strains were stored in 15% glycerol stocks at -80°C and were cultured on blood agar (BA) plates for 15–18 hours at 37°C the day prior to use. BA plates were prepared by the PathWest Media Preparation Unit in Shenton Park, Western Australia, Australia. *Staphylococcus aureus subsp*. *aureus* (ATCC 13709) was obtained from the American Type Culture Collection (ATCC) (Manassas, VA, USA). *Escherichia coli*, *Staphylococcus epidermidis*, *and Pseudomonas aeruginosa* species were clinical isolates obtained from patients with PD peritonitis, provided by Professor Tim Inglis, PathWest Laboratory Medicine WA. The identification of all isolates was confirmed using the MALDI Biotyper (Bruker, Victoria, Australia) and their antimicrobial susceptibilities with the automated Biomerieux Vitek 2 system.

### Antimicrobial Agents

Antimicrobial agents were selected based on current Australian and International Society for Peritoneal Dialysis (ISPD) guidelines [30, 31] and those recently used at our institution for the management of peritonitis. All agents were hospital pharmacy grade and doses were calculated on the assumption that antimicrobials were delivered in a standard 2L bag of dialysate.

Amoxicillin (Fisamox Amoxycillin, sodium powder for injection, 1g, Aspen Pharmacare, Australia) was prepared by adding 9.2 mL nuclease-free water to give a 100 mg/mL solution, and stored in 1 mL aliquots at -20°C. Amoxicillin was added to dialysate solutions at a final concentration of 150 mg/L.

Cefazolin (Cefazolin Sandoz, Cephazolin sodium powder for injection, 1g IM/IV, Sandoz) was prepared by adding 9.5 mL of nuclease-free water to obtain a 100 mg/mL solution, and stored in 1 mL aliquots at -20°C. Cefazolin was added to dialysate solutions at a final concentration of 500 mg/L.

Fluconazole (Fluconazole-Claris, Solution for IV Infusions, 100 mg in 50 mL, AFT Pharmaceuticals) was obtained as a 2 mg/mL solution and stored in 1 mL aliquots at room temperature and protected from light. Fluconazole was added to dialysate solutions at a final concentration of 100 mg/L.

Gentamicin (Gentamicin Injection BP, IV/IM use, 80 mg in 2 mL, Pfizer) was obtained as a 40 mg/mL solution and stored in 400 μL aliquots at -20°C. Gentamicin was added to dialysate solutions at a final concentration of 100 mg/L.

Vancomycin (Vancomycin Sandoz, Vancomycin Hydrochloride Powder for Injection, 500 mg IV) was reconstituted in 10 mL nuclease-free water to obtain a 50 mg/mL solution and stored in 1 mL aliquots at -20°C. Vancomycin was added to dialysate at a final concentration of 1 g/L. Vancomycin and Gentamicin were used in combination in dialysate solutions.

### tPA Chromogenic Assay

Dialysate solutions (dialysate alone, dialysate/tPA, dialysate/DNase or dialysate/tPA/DNase) were prepared and antimicrobials were added to aliquots of each of the four dialysate solutions at the final concentrations described above to give final volumes of 1 mL. After mixing well, 400 μL from each 1 mL tube was frozen at -20°C (T = 0 samples). The remaining samples were incubated at 37°C in a waterbath for 6 hours then frozen at -20°C (T = 6 samples). tPA activity was determined using a chromogenic tPA assay (Human tPA Chromogenic Activity Kit, CT1001, AssayPro USA) that measures the tPA-mediated cleavage of the zymogen plasminogen into the active serine protease plasmin, which digests fibrin [32]. Dialysate solutions (T = 0 and T = 6) were thawed at room temperature and diluted 1/500 in assay diluent to give a final in-assay tPA concentration of approximately 1.16 IU/mL, which corresponded to the mid-point of the standard curve. The assay was set up in duplicate according to the manufacturer’s instructions. Absorbance readings at 405 nm were measured using an EnVision 2102 Multilabel Reader (Perkin Elmer, Melbourne, Victoria, Australia). Following an initial background reading at A_405_ and a one hour incubation in a humid 37°C incubator, the plate was transferred to the Envision reader, maintained at 37°C and 25 hourly readings were taken at A_405_. A plate sealer was used at all times to prevent evaporation of samples. The percent tPA activity was determined as follows: [Calculated tPA Concentration (IU/mL) / Theoretical tPA Concentration (IU/mL)] x 100.

### DNase Activity

A DNA degradation assay was performed to determine the activity of DNase when exposed to dialysate solutions (dialysate alone, dialysate/tPA, dialysate/DNase or dialysate/tPA/DNase) with or without the presence of antimicrobials, or to the supernatant of dialysate solutions containing bacteria. The solutions (20 μL final volume) were tested for their ability to degrade 1 μg of DNA (vector pcDNA3, Life Technologies Australia, Mulgrave, Victoria, Australia) in 1 hour in a 37°C waterbath and results were visualized on a 2% agarose TAE gel using a 100 bp ladder (TrackIt 100 bp DNA ladder, Life Technologies) and ethidium bromide. *E*. *coli* DH5α cells (Life Technologies) containing pcDNA3 were grown up overnight at 37°C in LB broth containing 100 μg/mL Ampicillin and pcDNA3 was isolated using a Qiagen Plasmid Plus Midi kit. DNA concentrations were determined using a NanoDrop ND-1000 (Thermo Fisher Scientific USA) and adjusted to 1 μg/μL for DNase activity experiments.

### Viability Experiments

*Staphylococcus aureus*, *Staphylococcus epidermidis*, *Escherichia coli* and *Pseudomonas aeruginosa* were freshly grown from -80°C stocks on BA plates overnight at 37°C. Standardized suspensions were prepared in sterile saline to 10^10^ cfu/mL or 10^8^ cfu/mL, and diluted 1/10 in dialysate solutions (dialysate alone, dialysate/tPA, dialysate/DNase or dialysate/tPA/DNase) to give final concentrations of 10^9^ cfu/mL or 10^7^ cfu/mL of *S*. *aureus* or *E*. *coli*. All dialysate solutions containing bacteria were set up in triplicate, incubated in a shaking incubator at 37°C for 6 hours, and aliquots were taken for viability counts at 0, 2 and 6 hours. Serial dilutions were prepared in sterile PBS (Thermo Fisher Scientific, Scoresby, Victoria, Australia), 20 μL volumes were spotted in triplicate onto BA plates, allowed to dry, and plates were incubated inverted at 37°C for 15–18 hours. Colony counts were performed and the average viable count (cfu/mL) and standard deviation of each dialysate solution was calculated at each time point. Aliquots from the 10^7^ cfu/mL *S*. *aureus* and *E*. *coli* experiments at 0 and 6 hours were stored at -20°C, thawed at room temperature, centrifuged to pellet bacterial cells (13,300 rpm for 10 minutes at 4°C) and the supernatants were used in DNase activity experiments, as described above.

To determine whether tPA and DNase effect the activity of the antimicrobial agents used, the viability of *E*. *coli*, *Ps*. *aeruginosa*, *S*. *aureus* and *S*. *epidermidis* cultures with known sensitivities were measured in dialysate and dialysate/tPA/DNase solutions in the presence of different antibiotics at 0, 6 and 24 hours. *S*. *aureus* was tested at 10^7^ cfu/mL against Amoxicillin and Cefazolin, *S*. *epidermidis*, *E*. *coli* and, *Ps*. *aeruginosa* were tested at 10^7^ cfu/mL against Vancomycin/Gentamicin. All antibiotics were used at the concentrations described above. Viability controls were prepared using dialysate containing the appropriate antimicrobial in the absence of tPA/DNase. Triplicate cultures were prepared and viable counts determined as described previously.

### Animal Experiments

Animal experiments were performed on female CD1 mice aged 6–8 weeks (Animal Resources Centre, Perth, Australia). Approval for this work was granted by the Animal Ethics Committee of the University of Western Australia (Permit number 03/100/905). All treatments were administered by intraperitoneal injections using a 27-gauge needle. To determine the safety of tPA and DNase, mice received 150 μg tPA (n = 8), 50 μg DNase (n = 8), 150 μg tPA and 50 μg DNase (n = 8), or sterile saline (n = 7), all in 200 μL of saline. In the peritonitis model, mice received either tPA (100 μg) plus DNase (50 μg), with or without LPS (200 μg). Mice receiving LPS only (200 μg) or dialysate/PBS alone served as controls (n = 4 for all groups). All treatment groups contained 50 μL dialysate and, where necessary, total volumes were adjusted to 200 μL using sterile PBS. LPS rather than bacteria was used as an inflammatory stimulus, as previous experiments (unpublished) have demonstrated inconsistent responses following intraperitoneal administration of different bacterial species. The peritoneal cavity was lavaged with saline (1 mL) for assessment of ascites; pH (pH Indicator Strips, pH 6.5–10, Merck Millipore, Kilsyth, Victoria, Australia) and animals were weighed pre- and post-treatment as an additional measure of toxicity.

Mice were euthanized by cervical dislocation following anesthesia with methoxyflurane (Medical Developments Australia, Springvale, Victoria, Australia) 6 hours post treatment. A longitudinal midline incision was made to expose the peritoneum. The liver, left kidney, spleen and a section of peritoneal wall were removed and fixed in 4% paraformaldehyde (Sigma-Aldrich, New South Wales, Australia) for paraffin embedding. Sections (5 μm) were cut from multiple tissues of all mice, stained with Haemotoxylin and Eosin (Amber Scientific, Midvale, Western Australia, Australia) and reviewed by an independent histopathologist (DW) blinded to the treatment groups to assess for evidence of toxicity.

### Statistical Analyses

Statistical analyses were performed using Prism 6 for Mac OS X version 6.0d (GraphPad Software, La Jolla, California USA). Normality was assessed using the D’Agostino and Pearson omnibus test. Differences between groups were analysed by one-way analysis of variance (ANOVA) or independent t-tests for parametric data and Kruskal-Wallis or Wilcoxon matched-pairs signed rank test for non-parametric data. Statistical significance was defined as *p*<0.05.

## Results

### tPA and DNase are compatible with standard peritoneal dialysate solution

The compatibility of tPA and DNase with standard dialysis solution, in the presence and absence of antimicrobial agents was assessed by biochemical analysis of treated dialysate as well as visual inspection and spectrophotometric assessment for precipitation (at 650 nm) following 6 hours at 37°C. No changes in dialysate colour or consistency occurred, and there was no evidence of precipitate formation. The presence of tPA and DNase or antimicrobial agents did not affect the biochemical composition of the dialysate (S1 Fig).

### tPA and DNase retain biological activity when combined with antimicrobial agents used to treat PD peritonitis

**Fig 1:**
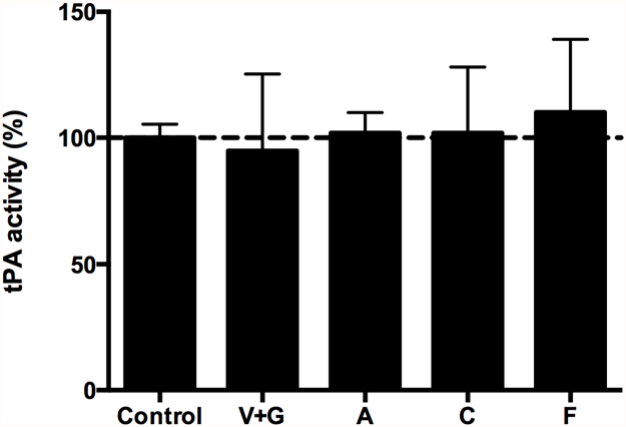
tPA activity in the presence of antimicrobial agents used for the treatment of PD peritonitis. The activity of tPA in the presence of different antimicrobial agents used for the treatment of PD peritonitis was assessed with reference to controls. Results are expressed as the mean and SD of percentage of activity in control wells from a representative experiment. Control = dialysate only, V+G = Vancomycin and Gentamicin, A = Amoxicillin, C = Cefazolin and F = Fluconazole.

**tPA Activity**. Dialysate solutions (dialysate alone, dialysate/tPA, dialysate/DNase or dialysate/tPA/DNase) were analysed for tPA activity using a chromogenic tPA assay that measures the ability of tPA to cleave plasminogen to plasmin. Conditions tested included dialysate alone, or dialysate solution containing antimicrobials used to treat PD peritonitis (Vancomycin and Gentamicin, Amoxicillin, Cefazolin or Fluconazole) after 6 hours shaking at 37°C. Samples were diluted 1/500 to give an expected tPA concentration of 1.16 IU/mL in the assay, which corresponds to 2900 IU/mL tPA in the original sample tubes (and the mid-point of the standard curve). tPA concentrations were measured after 6 hours in the presence or absence of antimicrobial agents (Fig. 1). All samples were tested in duplicate per the manufacturer’s instructions. No significant differences in tPA activity in samples containing antimicrobial agents were detected, when compared to controls (p>0.5).

**DNase Activity**. DNase activity was measured through the ability of DNase-containing samples to degrade a 1 μg sample of DNA of known size in 1 hour at 37°C. Two approaches were taken to assess whether antimicrobial agents themselves or common bacteria causing peritonitis might influence DNase activity: (1) Dialysate solutions ± DNase were incubated with antimicrobial agents, and (2) Dialysate solutions ± DNase were incubated in the presence of *S*. *aureus* (Gram positive) or *E*. *coli* (Gram negative).

**Fig 2:**
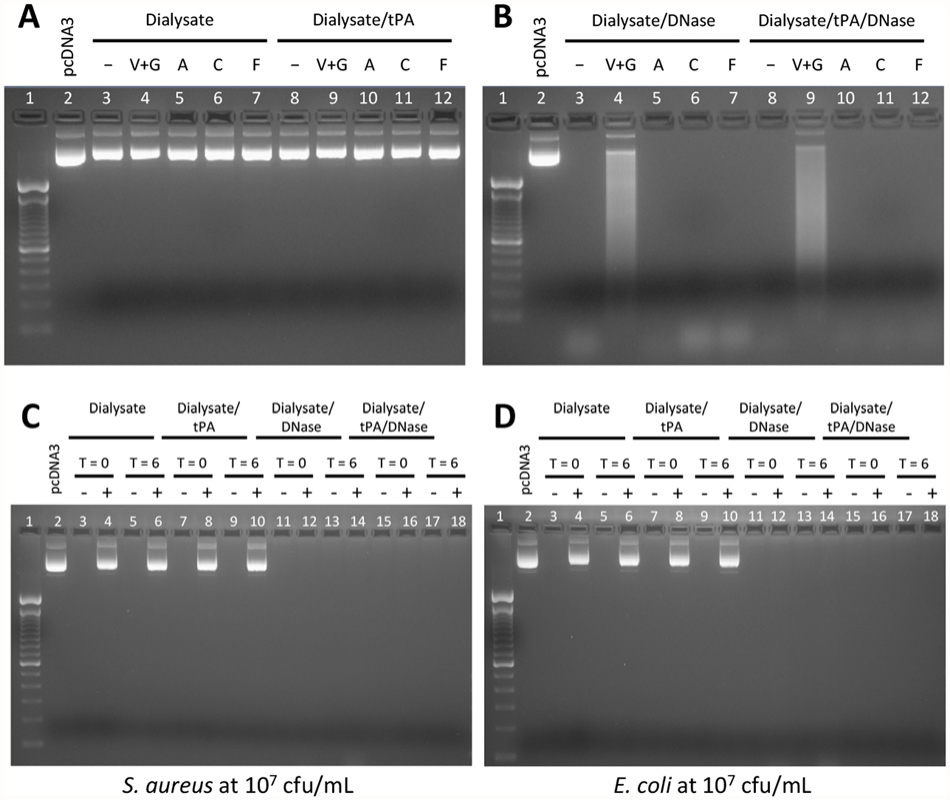
DNase activity after 6 hours incubation with tPA and antimicrobials or tPA and bacteria. The activity of DNase in the presence of different antimicrobial agents (Panels A & B) or bacteria (Panels C & D) was measured through digestion of 1 μg of pcDNA3 DNA. V+G = Vancomycin and Gentamicin, A = Amoxicillin, C = Cefazolin and F = Fluconazole. The effects of bacteria on DNase activity was assessed at baseline (T = 0) and following 6 hours incubation (T = 6) at 37°C. Samples were incubated with 5 μg/mL tPA and/or 2.5 μg/mL DNase, as applicable.

**Fig 3:**
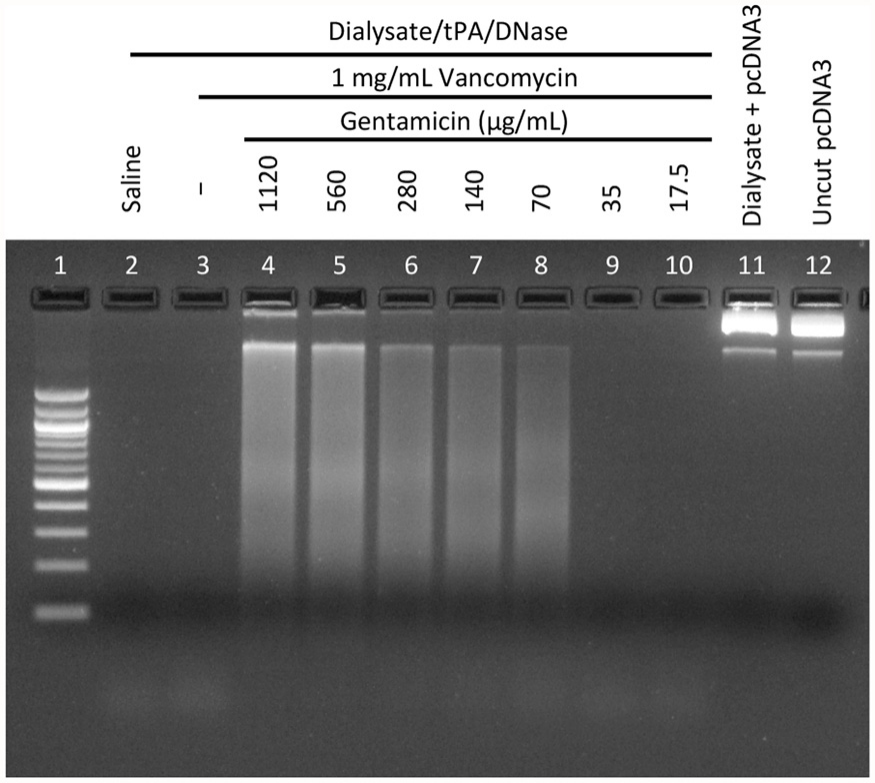
DNase activity in the presence of vancomycin and gentamicin. The effect of increasing concentrations of gentamicin on DNase activity was measured in the presence of a constant concentration of Vancomycin. Inhibition of DNase activity, visible as smearing (incomplete digestion) of DNA (Lanes 4–8), was evident at Gentamicin concentrations >35 μg/mL. Samples contained 5 μg/mL tPA and 2.5 μg/mL DNase, as appropriate.

No DNase activity was observed in the Dialysate alone or Dialysate/tPA groups, irrespective of the antimicrobial agents added (Fig. 2A). DNase activity was present in all samples from the dialysate/DNase or dialysate/tPA/DNase groups. However, the Vancomycin/Gentamicin samples in each group showed partial inhibition of DNase activity, evidenced by incomplete digestion of the DNA (Fig. 2B). Subsequent studies revealed that inhibition of DNase activity was caused by Gentamicin (Fig. 3) at concentrations above 35 μg/mL.

*S*. *aureus* or *E*. *coli* were added at 10^7^ cfu/mL to tubes containing dialysate alone, dialysate/tPA, dialysate/DNase or dialysate/tPA/DNase in a shaking incubator at 37°C for 6 hours. Aliquots were removed at 0 and 6 hours and bacterial cells pelleted by centrifugation. The supernatant was used to measure DNase activity through digestion of a known quantity of DNA as described above.. No DNase activity was detected in the dialysate alone or dialysate/tPA groups for *S*. *aureus* (Fig. 2C) or *E*. *coli* (Fig. 2D). In addition, exposure of dialysate to *S*. *aureus* (Fig. 2C) or *E*. *coli* (Fig. 2D) did not affect DNase activity, with complete digestion of DNA observed at 0 and 6 hours.

### tPA and DNase do not have any intrinsic antimicrobial properties

**Fig 4:**
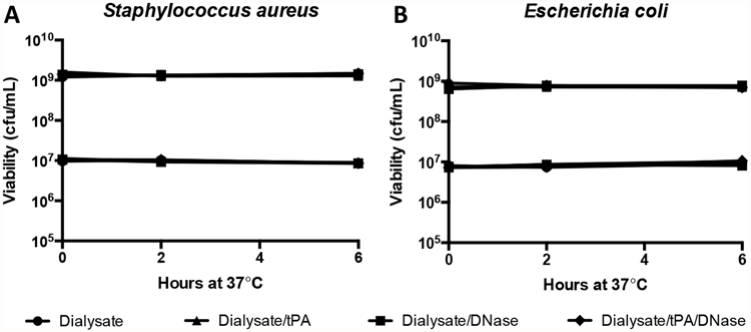
tPA and DNase do not directly affect the viability of *E*. *coli* and *S*. *aureus*.

To determine whether the addition of tPA and DNase directly to bacteria *ex vivo* influences their growth and survival, we next assessed bacterial viability by incubating two concentrations (10^7^ and 10^9^ cfu/mL) of *S*. *aureus* (Fig. 4A) and *E*. *coli* (Fig. 4B) in the presence or absence of tPA and DNase for up to 6 hours. Colony counts of serial dilutions of bacterial suspensions were performed to calculate the number of viable bacteria, which demonstrated that the addition of tPA and DNase had no effect on bacterial viability (p>0.1).

Bacterial viability in dialysate was measured through triplicate counts of spot dilutions on blood agar plates, using two concentrations of bacteria (10^7^ and 10^9^ cfu/mL). Samples contained 5 μg/mL tPA and/or 2.5 μg/mL DNase, as appropriate.

### tPA and DNase do not affect antimicrobial activity

**Fig 5:**
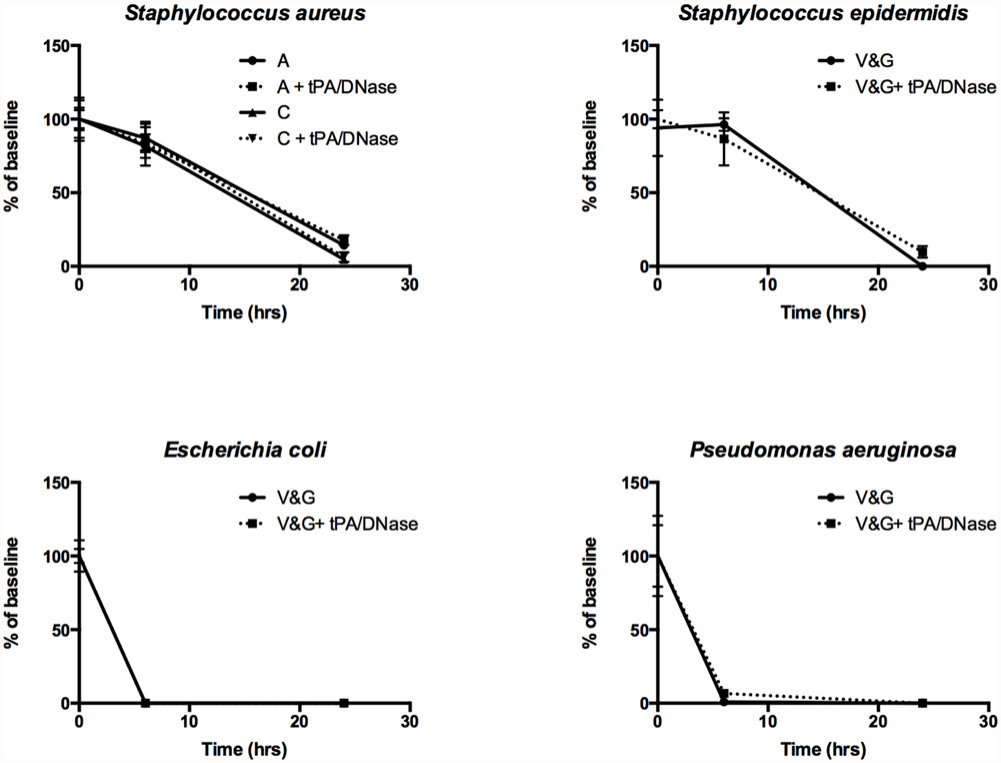
tPA and DNase do not influence antimicrobial activity. The effect of tPA and DNase on antimicrobial activity was assessed by measuring the survival of bacteria (with known sensitivity) to the antimicrobial agents over time in the presence or absence of tPA and DNase. All assessments were performed in triplicate and bacterial counts were calculated through spot counting of serial dilutions of bacteria. Data show is the mean number of cfu/mL at 6 and 24 hours as a % of baseline (error bars are the standard deviation). A = Amoxicillin, C = Cefazolin, V&G = Vancomycin and Gentamicin.

To assess whether the presence of tPA and DNase affects the antimicrobial activity of commonly used agents for the treatment of peritoneal dialysis-associated peritonitis, we incubated 10^7^ cfu/mL of *S*. *aureus*, *S*. *epidermidis*, *E*. *coli or Ps*. *aeruginosa* in the presence of antibiotics (Vancomycin and Gentamicin, Amoxicillin or Cefazolin) or antibiotics plus tPA and DNase, in dialysate. Bacterial viability was assessed through colony counts after 6 and 24 hours incubation and compared with baseline. No statistically significant changes in bacterial viability were observed in any of the groups or time points tested (Fig. 5A–5D).

### Intraperitoneal instillation of tPA, DNase or tPA + DNase in a murine model of peritonitis is not associated with evidence of toxicity

**Fig 6:**
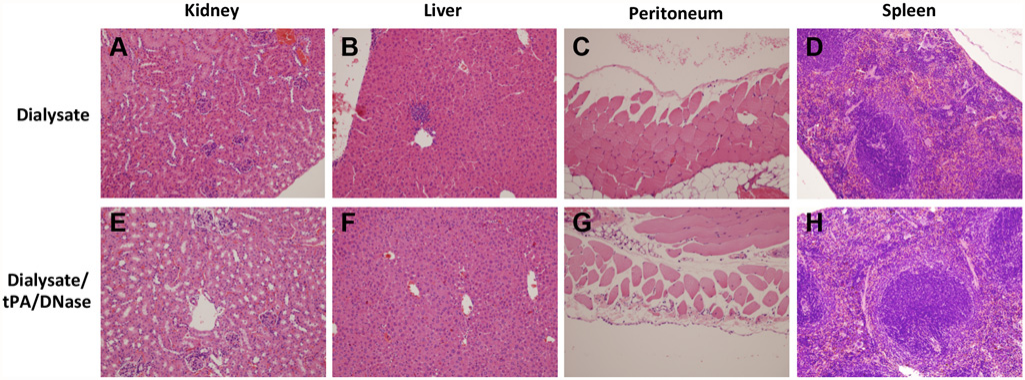
H&E sections of kidney, liver, peritoneum and spleen. Microscopic analysis of abdominal organs and peritoneum was performed following intraperitoneal administration of dialysate (A-D) or dialysate with tPA and DNase (E-H) in the presence or absence of LPS (data with LPS not shown). Samples contained 5 μg/mL tPA and 2.5 μg/mL DNase, as appropriate.

To determine whether tPA and/or DNase might cause toxicity in vivo, intraperitoneal instillation of tPA (5μg/mL) and/or DNase (2.5μg/mL) (the concentrations used in the pleural trial) was performed in a murine model of peritonitis. The peritoneum and intra-abdominal organs were macroscopically assessed at sacrifice and appeared normal in all mice, in the presence or absence of LPS. Microscopic assessment of the left kidney, liver, spleen and a section of the peritoneal wall did not reveal any abnormalities and there was no evidence of systemic toxicity or bleeding diatheses, as evidenced by haemorrhage within the organs or prolonged bleeding from puncture sites following administration of tPA and DNase, in the presence or absence of LPS (Fig. 6). Administration of tPA and/or DNase had no effect on mouse weights and did not induce ascites (no fluid was able to be aspirated at the time of lavage) or affect lavage fluid pH in any of the treatment groups (S2 Fig). Collectively these observations confirm that tPA and DNase do not appear to be toxic *in vivo*.

## Discussion

In this study we present pre-clinical data to support the feasibility of administering intraperitoneal tPA and DNase as an adjunctive therapy in the treatment of PD peritonitis. We have shown that tPA and DNase do not affect the appearance or biochemical composition of peritoneal dialysate solutions, and that with the exception of high dose Gentamicin, commonly used antimicrobial agents do not influence the activity of tPA and DNase. In addition tPA and DNase do not reduce the efficacy of commonly prescribed antimicrobial agents used to treat PD peritonitis patients.

Peritoneal dialysis is an important therapeutic option for the management of end stage kidney disease and there is a strong push to increase its utilization, due to its convenience and cost-effectiveness [33]. A major complication of peritoneal dialysis is the development of peritonitis, which is associated with many patients being unable to continue on PD and with significant morbidity and mortality [2, 8–11]. In a recent Australian study of 6639 patients receiving PD, peritonitis occurred on average once every 20 patient months on peritoneal dialysis [34].

Consistent with international guidelines [35], PD peritonitis in Western Australia is managed with broad-spectrum intraperitoneal antimicrobial therapy (Vancomycin and Gentamicin) with subsequent rationalisation of antibiotics once an organism is identified and sensitivities obtained. However, even with early administration of antibiotics, treatment failures are common [34, 36, 37].

The fibrinolytic system plays an integral role in the inflammatory response in peritonitis and intraperitoneal fibrinolytic agents have been used in the treatment of PD peritonitis to enhance antibiotic penetration [38]. Available data for urokinase suggests that use of this agent alone is ineffective as an adjunctive treatment in peritonitis [39–42], however several case reports have demonstrated the benefit of tPA in selected settings [19, 43]. As infected peritoneal fluid is DNA-rich these solutions are highly viscous, with viscosity related to the size of the DNA molecules present [44]. Cleavage of DNA can therefore facilitate the clearance of fluids containing high levels of DNA and may help disrupt biofilms [27].

Combining tPA and DNAse has been shown to improve clinical outcomes in pleural infections whereas either agent alone was ineffective [29]. However, in contrast to the pleural setting, where fibrinolytic agents and/or DNase are instilled alone into the pleural space, in peritoneal dialysis patients with peritonitis, these agents need to be compatible with, and retain activity, in the presence of dialysate and commonly used antimicrobials. We have demonstrated that tPA and DNase remain active in a standard dialysis solution containing common antimicrobial agents used for the empirical treatment of PD-peritonitis, except in the presence of high doses of gentamicin (>35 μg/mL), which reduce, but do not completely inhibit the activity of DNase (Fig. 3). We have also shown that DNase activity is unaffected by exposure to dialysate containing *S*. *aureus* or *E*. *coli* (the most commonly isolated Gram positive and Gram negative organisms) (Fig. 2), indicating that neither the bacterial cells nor any secreted products have detrimental effects on the ability of DNase to degrade DNA.

For tPA and DNase to be considered for incorporation into the treatment for PD peritonitis, these compounds must not increase bacterial viability or decrease antimicrobial efficacy. As shown, the viability of *S*. *aureus* or *E*. *coli* over a six-hour period, longer than most dialysis dwell times, was not increased in the presence of dialysate containing tPA and/or DNase (Fig. 4) and the addition of tPA and DNase to antimicrobial agents had no significant impact on their effectiveness against *S*. *aureus*, *S*. *epidermidis*, *E*. *coli* or *Ps*. *aeruginosa* (Fig. 5).

To confirm the *in vivo* safety of these agents when given intraperitoneally in combination, injections were performed in an established murine peritonitis model, as intraperitoneal inflammation may enhance peritoneal permeability and systemic absorption of agents [31, 45]. No adverse effects or signs of toxicity were observed locally or systemically, when tPA and DNase were administered (Fig. 6).

Our study has several limitations. We were unable to test all possible combinations of antimicrobial agents and microbial species commonly encountered in clinical practice, and did not examine the impact of biocompatible vs. non-biocompatible peritoneal dialysis solutions. Given the absence of ascitic fluid in our *in vivo* model, we were also unable to assess the impact of local cytokine production on the activity of tPA and DNase, which may be important, as high levels of plasminogen activator inhibitor type 1 (PAI-1) have been reported in patients with peritonitis [46, 47]. This may necessitate the use of higher doses of tPA or more frequent administration. In addition, due to restrictions imposed by the animal ethics committee, repeated injections of tPA/DNase in the LPS peritonitis model could not be performed. However, extensive clinical experience of repeated dosing in the setting of pleural infections has demonstrated that treatment is safe and effective [48]. Of interest will be further assessment of tPA and DNase in the settings of relapsing, refractory and fungal peritonitis, where outcomes are particular poor [31, 49, 50].

The significant complications associated with PD peritonitis, including reduced modality and patient survival as well as the costs to health care systems, highlights the need for new and more effective treatment options. Incorporating tPA and DNase into a standard antibiotic treatment regimes for PD peritonitis may improve patient outcomes by increasing antibiotic penetration, and reducing adhesions, biofilms and fluid viscosity, which may contribute to ultrafiltration failures and recurrent and refractory infections.

## Supporting Information

S1 Fig Effects of tPA and DNase and antimicrobial agents on dialysate composition. Dialysate composition following 6 hours incubation with tPA/DNase or various antimicrobial agents is shown. No difference in any of the parameters was detected (p>0.2 for all).
(TIF)

S2 Fig Effects of intraperitoneal instillation of tPA and DNase. A) Animal weights following intraperitoneal administration at baseline and 6 hours. Weights decreased significantly in animals treated with LPS (p = 0.01 and 0.04 in the absence or presence of tPA/DNase) however there were no differences between dialysate and tPA/DNase treated animals. B) Volume of intraperitoneal fluid obtained post-instillation. C) Lavage fluid pH.
(TIF)

## References

[1] Eckardt KU, Coresh J, Devuyst O, Johnson RJ, Kottgen A, et al (2013) Evolving importance of kidney disease: from subspecialty to global health burden. Lancet 382: 158–169. 10.1016/S0140-6736(13)60439-0 23727165

[2] Hurst K, Clayton P, McDonald S (2012) Annual Report Summary In: S. McDonald, P. Clayton and K. Hurst, editors. ANZDATA Registry Report 2012. Adelaide, South Australia pp. XXIII.

[3] Jager KJ, Korevaar JC, Dekker FW, Krediet RT, Boeschoten EW (2004) The effect of contraindications and patient preference on dialysis modality selection in ESRD patients in The Netherlands. American journal of kidney diseases: the official journal of the National Kidney Foundation 43: 891–899. 15112180 10.1053/j.ajkd.2003.12.051

[4] Lamping DL, Constantinovici N, Roderick P, Normand C, Henderson L, et al (2000) Clinical outcomes, quality of life, and costs in the North Thames Dialysis Study of elderly people on dialysis: a prospective cohort study. Lancet 356: 1543–1550. 11075766 10.1016/S0140-6736(00)03123-8

[5] Boateng EA, East L (2011) The impact of dialysis modality on quality of life: a systematic review. J Ren Care 37: 190–200. 10.1111/j.1755-6686.2011.00244.x 22035363

[6] Ghaffari A, Kalantar-Zadeh K, Lee J, Maddux F, Moran J, et al (2013) PD First: Peritoneal Dialysis as the Default Transition to Dialysis Therapy. Semin Dial. 10.1111/sdi.12125 24102745

[7] Brown F, Liu WJ, Kotsanas D, Korman TM, Atkins RC (2007) A quarter of a century of adult peritoneal dialysis-related peritonitis at an Australian medical center. Peritoneal dialysis international: journal of the International Society for Peritoneal Dialysis 27: 565–574. 17704449

[8] Boudville N, Kemp A, Clayton P, Lim W, Badve SV, et al (2012) Recent peritonitis associates with mortality among patients treated with peritoneal dialysis. Journal of the American Society of Nephrology: JASN 23: 1398–1405. 10.1681/ASN.2011121135 22626818 PMC3402287

[9] Mastrosimone S, Virga G, Gastaldon F, da PA, Bonadonna A (2002) Low peritonitis rate leads to high patient survival and technique success: the first five years of a peritoneal dialysis program. Perit Dial Int 22: 91–93. 11929152

[10] Munoz de Bustillo E, Borras F, Gomez-Roldan C, Perez-Contreras FJ, Olivares J, et al (2011) Impact of peritonitis on long-term survival of peritoneal dialysis patients. Nefrologia: publicacion oficial de la Sociedad Espanola Nefrologia 31: 723–732. 10.3265/Nefrologia.pre2011.Oct.10987 22130289

[11] Kumar VA, Sidell M, Yang WT, Jones JP (2013) Predictors of Peritonitis, Hospital Days, and Technique Survival for Peritoneal Dialysis Patients in a Managed Care Setting. Perit Dial Int. 10.3747/pdi.2012.00165 PMC3968102 24084841

[12] Ahrenholz DH, Simmons RL (1980) Fibrin in peritonitis. I. Beneficial and adverse effects of fibrin in experimental E. coli peritonitis. Surgery 88: 41–47. 6992321

[13] Hanlon GW, Denyer SP, Hodges NA, Brant JA, Lansley AB, et al (2004) Biofilm formation and changes in bacterial cell surface hydrophobicity during growth in a CAPD model system. The Journal of pharmacy and pharmacology 56: 847–854. 15233862 10.1211/0022357023817

[14] Pihl M, Davies JR, Johansson AC, Svensater G (2013) Bacteria on catheters in patients undergoing peritoneal dialysis. Perit Dial Int 33: 51–59. 10.3747/pdi.2011.00320 22855889 PMC3598254

[15] Read RR, Eberwein P, Dasgupta MK, Grant SK, Lam K, et al (1989) Peritonitis in peritoneal dialysis: bacterial colonization by biofilm spread along the catheter surface. Kidney Int 35: 614–621. 2709667 10.1038/ki.1989.30

[16] Sepandj F, Ceri H, Gibb AP, Read RR, Olson M (2003) Biofilm infections in peritoneal dialysis-related peritonitis: comparison of standard MIC and MBEC in evaluation of antibiotic sensitivity of coagulase-negative staphylococci. Perit Dial Int 23: 77–79. 12691511

[17] Dasgupta MK, Kowalewska-Grochowska K, Larabie M, Costerton JW (1991) Catheter biofilms and recurrent CAPD peritonitis. Adv Perit Dial 7: 165–168. 1680417

[18] Marcus RJ, Post JC, Stoodley P, Hall-Stoodley L, McGill RL, et al (2008) Biofilms in nephrology. Expert Opin Biol Ther 8: 1159–1166. 10.1517/14712598.8.8.1159 18613767

[19] Duch JM, Yee J (2001) Successful use of recombinant tissue plasminogen activator in a patient with relapsing peritonitis. American journal of kidney diseases: the official journal of the National Kidney Foundation 37: 149–153. 11136181 10.1016/s0272-6386(01)80069-x

[20] Worland MA, Radabaugh RS, Mueller BA (1998) Intraperitoneal thrombolytic therapy for peritoneal dialysis—associated peritonitis. Annals of Pharmacotherapy 32: 1216–1220. 9825089 10.1345/aph.16153

[21] Murphy G, Tzamaloukas AH, Eisenberg B, Gibel LJ, Avasthi PS (1991) Intraperitoneal thrombolytic agents in relapsing or persistent peritonitis of patients on continuous ambulatory peritoneal dialysis. Int J Artif Organs 14: 87–91. 2037395

[22] Shea M, Hmiel SP, Beck AM (2001) Use of tissue plasminogen activator for thrombolysis in occluded peritoneal dialysis catheters in children. Advances in peritoneal dialysis Conference on Peritoneal Dialysis 17: 249–252. 11510286

[23] Zorzanello MM, Fleming WJ, Prowant BE (2004) Use of tissue plasminogen activator in peritoneal dialysis catheters: a literature review and one center’s experience. Nephrology nursing journal: journal of the American Nephrology Nurses’ Association 31: 534–537. 15518255

[24] Hall-Stoodley L, Nistico L, Sambanthamoorthy K, Dice B, Nguyen D, et al (2008) Characterization of biofilm matrix, degradation by DNase treatment and evidence of capsule downregulation in Streptococcus pneumoniae clinical isolates. BMC Microbiol 8: 173 10.1186/1471-2180-8-173 18842140 PMC2600794

[25] Thomas VC, Thurlow LR, Boyle D, Hancock LE (2008) Regulation of autolysis-dependent extracellular DNA release by Enterococcus faecalis extracellular proteases influences biofilm development. J Bacteriol 190: 5690–5698. 10.1128/JB.00314-08 18556793 PMC2519388

[26] Izano EA, Amarante MA, Kher WB, Kaplan JB (2008) Differential roles of poly-N-acetylglucosamine surface polysaccharide and extracellular DNA in Staphylococcus aureus and Staphylococcus epidermidis biofilms. Appl Environ Microbiol 74: 470–476. 18039822 10.1128/AEM.02073-07 PMC2223269

[27] Eckhart L, Fischer H, Barken KB, Tolker-Nielsen T, Tschachler E (2007) DNase1L2 suppresses biofilm formation by Pseudomonas aeruginosa and Staphylococcus aureus. Br J Dermatol 156: 1342–1345. 17459041 10.1111/j.1365-2133.2007.07886.x

[28] Nemoto K, Hirota K, Murakami K, Taniguti K, Murata H, et al (2003) Effect of Varidase (streptodornase) on biofilm formed by Pseudomonas aeruginosa. Chemotherapy 49: 121–125. 12815204 10.1159/000070617

[29] Rahman NM, Maskell NA, West A, Teoh R, Arnold A, et al (2011) Intrapleural use of tissue plasminogen activator and DNase in pleural infection. The New England journal of medicine 365: 518–526. 10.1056/NEJMoa1012740 21830966

[30] Walker A, Bannister K, George C, Mudge D, Yehia M, et al (2013) KHA-CARI Guideline: Peritonitis Treatment and Prophylaxis. Nephrology (Carlton). 10.1111/nep.12152 23944845

[31] Li PK-T, Szeto CC, Piraino B, Bernardini J, Figueiredo AE, et al (2010) Peritoneal Dialysis-Related Infections Recommendations: 2010 Update. Peritoneal Dialysis International 30: 393–423. 10.3747/pdi.2010.00049 20628102

[32] Vassalli JD, Sappino AP, Belin D (1991) The plasminogen activator/plasmin system. The Journal of clinical investigation 88: 1067–1072. 1833420 10.1172/JCI115405 PMC295552

[33] Davies SJ (2013) Peritoneal dialysis[mdash]current status and future challenges. Nat Rev Nephrol 9: 399–408. 10.1038/nrneph.2013.100 23689122

[34] Ghali JR, Bannister KM, Brown FG, Rosman JB, Wiggins KJ, et al (2011) Microbiology and outcomes of peritonitis in Australian peritoneal dialysis patients. Peritoneal dialysis international: journal of the International Society for Peritoneal Dialysis 31: 651–662. 10.3747/pdi.2010.00131 21719685

[35] Li PK, Szeto CC, Piraino B, Bernardini J, Figueiredo AE, et al (2010) Peritoneal dialysis-related infections recommendations: 2010 update. Perit Dial Int 30: 393–423. 10.3747/pdi.2010.00049 20628102

[36] Kim GC, Korbet SM (2000) Polymicrobial peritonitis in continuous ambulatory peritoneal dialysis patients. American journal of kidney diseases: the official journal of the National Kidney Foundation 36: 1000–1008. 11054357 10.1053/ajkd.2000.19102

[37] Johnson D (2006) Peritoneal dialysis ANZDATA Registry Report: 2006. Adelaide, Australia pp. 88–102.

[38] Worland MA, Radabaugh RS, Mueller BA (1998) Intraperitoneal thrombolytic therapy for peritoneal dialysis-associated peritonitis. The Annals of pharmacotherapy 32: 1216–1220. 9825089 10.1345/aph.16153

[39] Innes A, Burden RP, Finch RG, Morgan AG (1994) Treatment of resistant peritonitis in continuous ambulatory peritoneal dialysis with intraperitoneal urokinase: a double-blind clinical trial. Nephrology, dialysis, transplantation: official publication of the European Dialysis and Transplant Association—European Renal Association 9: 797–799. 7970121

[40] Gadallah MF, Tamayo A, Sandborn M, Ramdeen G, Moles K (2000) Role of intraperitoneal urokinase in acute peritonitis and prevention of catheter loss in peritoneal dialysis patients. Advances in peritoneal dialysis Conference on Peritoneal Dialysis 16: 233–236. 11045301

[41] Tong MK, Leung KT, Siu YP, Lee KF, Lee HK, et al (2005) Use of intraperitoneal urokinase for resistant bacterial peritonitis in continuous ambulatory peritoneal dialysis. Journal of nephrology 18: 204–208. 15931649

[42] Wiggins KJ, Craig JC, Johnson DW, Strippoli GF (2008) Treatment for peritoneal dialysis-associated peritonitis. Cochrane Database Syst Rev: CD005284 10.1002/14651858.CD005284.pub2 18254075

[43] Zorzanello MM, Fleming WJ, Prowant BE (2004) Use of tissue plasminogen activator in peritoneal dialysis catheters: a literature review and one center’s experience. Nephrol Nurs J 31: 534–537. 15518255

[44] Shak S, Capon DJ, Hellmiss R, Marsters SA, Baker CL (1990) Recombinant human DNase I reduces the viscosity of cystic fibrosis sputum. Proc Natl Acad Sci U S A 87: 9188–9192. 2251263 10.1073/pnas.87.23.9188 PMC55129

[45] McIntosh ME, Smith WG, Junor BJ, Forrest G, Brodie MJ (1985) Increased peritoneal permeability in patients with peritonitis undergoing continuous ambulatory peritoneal dialysis. European journal of clinical pharmacology 28: 187–191. 3987798 10.1007/BF00609690

[46] Ince A, Eroglu A, Tarhan O, Bülbül M (2002) Peritoneal fibrinolytic activity in peritonitis. American journal of surgery 183: 67–69. 11869706 10.1016/s0002-9610(01)00850-9

[47] de Boer AW, Levi M, Reddingius RE, Willems JL, van den Bosch S, et al (1999) Intraperitoneal hypercoagulation and hypofibrinolysis is present in childhood peritonitis. Pediatric nephrology 13: 284–287. 10454774 10.1007/s004670050609

[48] Piccolo F, Pitman N, Bhatnagar R, Popowicz N, Smith NA, et al (2014) Intrapleural tPA and DNase for Pleural Infection: an effective and safe alternative to surgery. Annals of the American Thoracic Society. 10.1513/AnnalsATS.201407-329OC 25296241

[49] Miles R, Hawley CM, McDonald SP, Brown FG, Rosman JB, et al (2009) Predictors and outcomes of fungal peritonitis in peritoneal dialysis patients. Kidney Int 76: 622–628. 10.1038/ki.2009.202 19516241

[50] Ghali JR, Bannister KM, Brown FG, Rosman JB, Wiggins KJ, et al (2011) Microbiology and Outcomes of Peritonitis in Australian Peritoneal Dialysis Patients. Peritoneal Dialysis International 31: 651–662. 10.3747/pdi.2010.00131 21719685

