# Supplementary figures and images for: Preclinical Assessment of Adjunctive tPA and DNase for Peritoneal Dialysis Associated Peritonitis
Source: PLoS One. 2015 Mar 5;10(3):e0119238. doi: 10.1371/journal.pone.0119238 (PMC4351066; doi:10.1371/journal.pone.0119238)

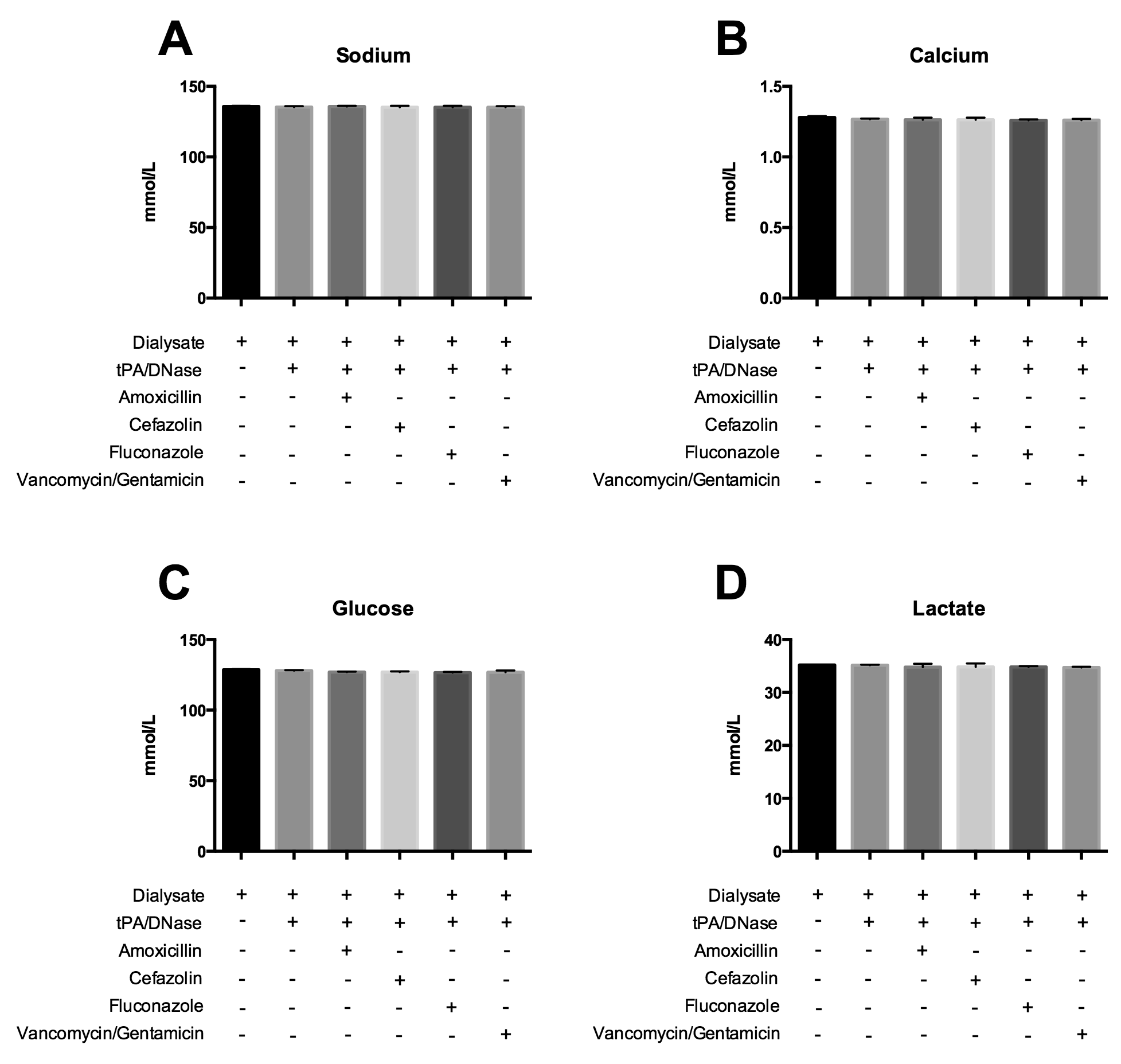

Supplement: S1 Fig — Dialysate composition following 6 hours incubation with tPA/DNase or various antimicrobial agents is shown. No difference in any of the parameters was detected (p>0.2 for all). (TIF) [file pone.0119238.s001.tif]

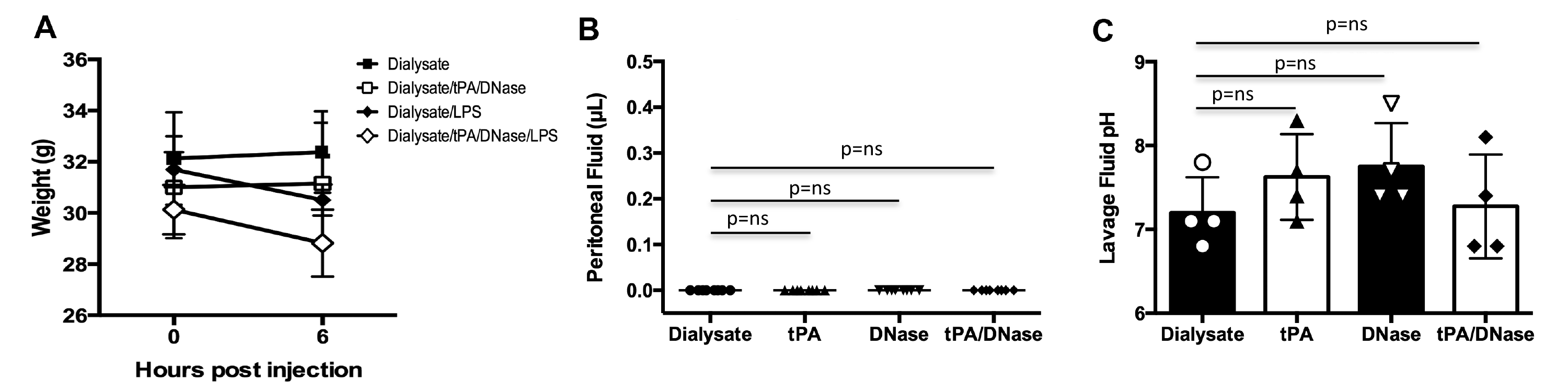

Supplement: S2 Fig — A) Animal weights following intraperitoneal administration at baseline and 6 hours. Weights decreased significantly in animals treated with LPS (p = 0.01 and 0.04 in the absence or presence of tPA/DNase) however there were no differences between dialysate and tPA/DNase treated animals. B) Volume of intraperitoneal fluid obtained post-instillation. C) Lavage fluid pH. (TIF) [file pone.0119238.s002.tif]
